# Sustaining the Yield of Maize, Blackgram, Greengram, Groundnut, Cotton, Sugarcane, and Coconut through the Application of Nutrients and Plant Growth Regulator Mixture

**DOI:** 10.3390/plants13111549

**Published:** 2024-06-04

**Authors:** Senthil Alagarswamy, Kalarani M. Karuppasami, Maduraimuthu Djanaguiraman, Prasad B. R. Venugopal, Sritharan Natarajan, Sivakumar Rathinavelu, Vijayalakshmi Dhashnamurthi, Ravichandran Veerasamy, Boominathan Parasuraman

**Affiliations:** 1 Department of Crop Physiology, Tamil Nadu Agricultural University, Coimbatore 641003, India; senthil.a@tnau.ac.in (S.A.); prasadvenugopal@gmail.com (P.B.R.V.); sivakumar.r@tnau.ac.in (S.R.); vijayalakshmi.d@tnau.ac.in (V.D.); ravi.v@tnau.ac.in (R.V.); boominathan.p@tnau.ac.in (B.P.); 2 Directorate of Crop Management, Tamil Nadu Agricultural University, Coimbatore 641003, India; 3 Department of Rice, Tamil Nadu Agricultural University, Coimbatore 641003, India; sritharan.n@tnau.ac.in

**Keywords:** field crops, nutrients, physiological traits, plant growth regulators, yield

## Abstract

The foliar application of nutrients and plant growth regulators (PGRs) at critical crop growth periods can improve the yield of field crops. Hence, the present study was conducted to quantify the effects of the combined application of nutrients and PGRs (crop-specific formulation) on maize, blackgram, greengram, groundnut, cotton, sugarcane, and coconut yield. In all the crops except coconut, the treatments included (i) a foliar spray of crop-specific nutrients and PGR combinations and (ii) an unsprayed control. In coconut, the treatments included (i) the root feeding of coconut-specific nutrients and PGR combinations and (ii) an untreated control. Crop-specific nutrient and PGR formulations were sprayed, namely, Tamil Nadu Agricultural University (TNAU) maize maxim 1.5% at the tassel initiation and grain-filling stages of maize, TNAU pulse wonder 1.0% at the peak flowering stage of green gram and black gram, TNAU groundnut-rich 1.0% at the flowering and pod-filling stages of groundnut, TNAU cotton plus 1.25% at the flowering and boll development stages of cotton, and TNAU sugarcane booster 0.5% at 45 days after planting (DAP), 0.75% at 60 DAP, and 1.0% at 75 DAP of sugarcane. The results showed that the foliar application of TNAU maize maxim, TNAU pulse wonder, TNAU groundnut-rich, TNAU cotton plus and TNAU sugarcane booster and the root feeding of TNAU coconut tonic increased the yield of maize, pulses, groundnut, cotton, sugarcane, and coconut, resulting in higher economic returns.

## 1. Introduction

The green revolution during 1960s boosted the yields of key staple food crops to meet the calorie needs of the growing world population [1]. Developing countries experienced an exceptional period of growth in food productivity over the past 50 years. Between 1960 and 2000, yields of crops in all developing countries increased by 208% for wheat, 109% for rice, 157% for maize, 78% for potatoes, and 36% for cassava [2]. However, by 2050, global agricultural production must increase by 110–200% to provide food security for the 8.2 billion people [3]. The yield of any field crop can be increased by improving its yield components [4]. Enhanced flowering, fruiting, and seed setting are important parameters directly correlated with crop yield, playing a major role in crop production. Maize (*Zea maize* L.) is a major food crop grown widely after rice (*Oryza sativa* L.) and wheat (*Triticum aestivum* L.). Similarly, among the pulses, greengram (*Vigna radiata* L.) and blackgram (*Vigna mungo* L.) are the most important crops in terms of meeting protein demands. Groundnut (*Arachis hypogaea* L.), cotton (*Gossypium hirsutum* L.), and sugarcane (*Saccharum officinarum* L.) are important oilseed, fibre, and sugar crops, respectively grown across the globe. The yield of these crops must be improved to sustain food production and for nutritional security. Hence, this study focused on the development of crop-specific boosters by considering the nutrient demand and plant growth regulators (PGR) requirement of maize, pulses, groundnut, cotton, sugarcane, and coconut at their critical growth stages in order to improve the yield per unit area.

Nutrients and plant growth regulators play an important role in the growth and development of plants. Hence, balanced crop nutrient management is essential during critical growth stages in order to improve yield. The foliar application of nutrients like nitrogen (N), phosphorus (P), potassium (K), boron (Bo), zinc (Zn), iron (Fe), and magnesium (Mg) at specific crop growth stages can increase grain yield. One of the most important mineral elements that plants require in larger amounts is nitrogen, a constituent of several plant cell components that plays a significant role in the synthesis of chlorophyll and enzymes in the photosynthetic process [5]. Next to nitrogen, phosphorus is also an important component of ATP, playing a crucial role in physiological and biochemical processes such as cellular respiration, nucleic acid synthesis, and root development in plants. Potassium is the third major nutrient that plays an important role in the opening and closing of stomata [6]. Additionally, it acts as an activator of the numerous enzymes involved in the synthesis of starch, protein, and ATP. Sulphur is involved in the synthesis of amino acids and fatty acids, activates the Co-A enzymes, and is also responsible for the oil content in oil seed crops like groundnut.

Micronutrients such as iron and zinc are involved in various metabolic activities like the synthesis of DNA and chlorophyll, photosynthesis, and respiration. They also act as activators of several enzymes in plants [7]. Even though iron is an abundant element in nature, plants cannot acquire it from its sources due to its immobile nature. Hence, its deficiency is common in many plants, causing interveinal chlorosis in young leaves. Kazemi [8] stated that the foliar application of zinc and iron increased plant height in tomato. In plant metabolism, boron plays an essential role in enzymatic activity, cell division, the translocation of carbohydrates, calcium and potassium uptake, and protein synthesis. It is one of the important micronutrients involved in maintaining the structural and functional integrity of plant cell membranes and translocating photosynthates to sink tissues [9]. It also facilitates the uptake and translocation of other micro- and macronutrients throughout the plant’s life cycle. An adequate level of boron facilitates effective pollination and a higher seed set. Ultimately, it enhances pod and seed development as it is involved in plant reproduction and pollen grain germination [10,11]. The combination of boron and zinc micronutrients also enhances the number of bolls and sympodial branches during cotton cultivation [12,13]. According to Takano et al. [14], boron is immobile in plants and accumulates in older leaves. In such cases, the foliar application of boron will help the plants to rectify the deficiency of boron in younger leaves.

The availability of essential macro- and micronutrients such as P, Fe and Mn is limited to the plants due to an imbalance of soil nutrients, the low soil moisture content observed with increased temperature, and other factors that prevent the effective absorption of these nutrients by plant roots [15]. In such situations, the foliar application of nutrients is more economical and effective than the soil-based application. The foliar application of macro- and micronutrients during the critical stages of plant growth sees them readily absorbed by plants through leaves, making nutrients quickly available to plants [16] and rectifying the hidden hunger for nutrients and explicit nutrient deficiencies seen in later growth stages, thereby enhancing crop yields.

Like nutrients, PGRs are small signalling molecules that can modulate the growth and development of crops through cell division and elongation and also through altered metabolism. Based on their physiological functions, PGRs were classified into two broad categories: growth-promoting (auxins, gibberellins, and cytokinin) and growth-inhibiting hormones (abscisic acid and ethylene). In general, PGRs are widely known for improving nutrient uptake, stress tolerance, membrane stability, and photoassimilate partitioning. Likewise, commercial PGRs such as 1-naphthaleneacetic acid (NAA), epibrassinolide, gibberellic acid, putrescine, salicylic acid, and triacontanol are frequently used in crop production [17]. According to Hanaa and Safaa [18], the foliar application of NAA increased the spike length and grain yield of wheat.

Similarly, in mustard, the application of cycocel (CCC), an anti-gibberellin, shifted the vegetative phase to the reproductive stage, resulting in increased yield [19]. Boll shedding is one of the main factors contributing to yield loss in cotton, a problem that the application of NAA can mitigate. Due to its function in cell division and elongation, this causes increased fruiting branches and bolls [20]. Additionally, it has been discovered that salicylic acid improves physiological and biochemical functions, leading to improved crop growth and production [21]. The application of NAA has been associated with higher seed cotton production [22]. Haifaa et al. [23] reported that the exogenous application of phytohormones in rice led to an increase in spikelet fertility. Kavitha et al. [24] reported that the foliar application of TNAU pulse wonder at a 2% concentration during the flowering and pod formation stages improved the number of flowers, given in plant^−1^, and green pod yield, given in ha^−1^. Similarly, Ashraf et al. [25] reported that the foliar application of TNAU pulse wonder improved the seed yield in pulses. El-Fouly et al. [26] reported that the foliar application of nutrients to maize resulted in increased grain yield. Among the nutrients sprayed, the combination of nitrogen and potassium significantly increased the grain yield compared to the use of individual nutrients [27]. A field trial on groundnut showed that the foliar application of a nutrient and plant growth regulator mixture significantly increased the groundnut yield, and the impact of the nutrient and plant growth regulator mixture was high in new varieties of groundnut [28].

Crops like greengram, blackgram, and groundnut are indeterminate, and this character causes continuous competition for photoassimilate between the vegetative and reproductive sinks, resulting in the abscission of flowers and pods. In this situation, a foliar spray of nutrients and PGRs will modify the reproductive physiology, resulting in higher yield. Similarly, in maize, the number of seeds per cob is under the influence of boron and auxin concentrations during the anthesis stage. Therefore, a foliar spray of nutrients and PGR combinations containing boron and NAA might improve the seed numbers, given in cob^−1^, seen in maize. In sugarcane, the economic yield is cane, and the cane weight can be improved by increasing the cane length. Based on the above, crop-specific nutrient mixtures and PGR formulations were developed. However, the effect of the combined application of nutrients and PGRs in crops was not studied in detail. Hence, the present study was conducted to quantify the effects of the combined application of nutrients and PGRs (crop-specific formulation) on the yield of maize, blackgram, greengram, groundnut, cotton, sugarcane, and coconut.

## 2. Materials and Methods

All the field experiments were conducted at the Tamil Nadu Agricultural University, Coimbatore—641003, Tamil Nadu, India during the period of 2020–2022. The experimental site is located at 11.01° N latitude and 76.93° E longitude, at an altitude of 426.7 m above MSL. The weather during the crop growing season is presented in Supplementary Table S1. During the crop growing season, the air temperature, rainfall and relative humidity were optimum, and no extreme events were observed.

Basic information about the name of the crop and variety, soil chemical composition, texture, irrigation scheduling, the quantity of fertilizer applied, field size, spacing between the rows and plants, and number of plants, determined in plot^−1^, is given in Table 1. The methodology followed for soil nutrient and protein estimation is presented in Table 2. Similarly, the stage of spray on the BBCH scale and concentration of TNAU crop-specific booster applied to each crop are presented in Table 3. The composition of the TNAU crop-specific crop booster is presented in Table 4.

### 2.1. Maize

A field experiment was carried out at the Eastern block farmland, Tamil Nadu Agricultural University (TNAU), Coimbatore, to evaluate the efficacy of TNAU Maize Maxim, a crop-specific mixture of nutrients and plant growth regulators, when applied to maize. The maize variety Co 8 was used in this experiment. The crop was sown at a spacing of 60 × 45 cm. The experiment was carried out in an area of 4000 m^2^ with a plot size of 500 m^2^, containing 3300 plants, by adopting a randomized block design with three replications. Plant protection measures were followed as per the Crop Production Guide [34] of Tamil Nadu state, India.

#### 2.1.1. Treatment Imposition

TNAU maize maxim is a crop booster with a mixture of nutrients and growth regulators. Two treatments, *viz*., a control and the foliar spraying of TNAU maize maxim 1.5%, were imposed at tassel initiation and grain-filling stages.

#### 2.1.2. Traits Recorded

Five plants from each replication were tagged at the physiological maturity stage in order to record data. The length of the cob (after husk removal) and cob diameter were measured for the fresh cob. The cob length was measured with the help of a ruler, and the average cob length was computed. The length and diameter of the cob were expressed in cm. The number of kernel rows, given as cob^−1^, and the number of kernels per row in a cob were counted manually. The kernel cob^−1^ was calculated by multiplying the number of rows, cob^−1^, by the number of kernel row^−1^. The weight of the cob was arrived at via weighing and the result was expressed in g cob^−1^. The kernels were air-dried, and the 100-grain weight from each treatment was recorded and expressed in g. The kernel yield ha^−1^ was calculated using the mean plot yield and expressed in kg ha^−1^.

### 2.2. Blackgram and Greengram

A field experiment was conducted at the Eastern block farmland, TNAU, Coimbatore, to evaluate the effect of TNAU pulse wonder on the yield of blackgram and greengram. The varieties used were Co 8 for greengram and Co 8 for blackgram. The experiment was carried out in an area of 4000 m^2^ with a plot size of 500 m^2^ containing 16,000 plants by adopting a randomized block design with three replications. Greengram and blackgram were sown in separate fields in ridges and furrows, with an area of 4000 m^2^ planted for each crop. Crop husbandry was the same for both crops. Plant protection measures were followed as per those of the Crop Production Guide [34].

#### 2.2.1. Treatment Imposition

Two treatments were imposed on each crop. The first treatment was an unsprayed control, and the second treatment was a foliar spray of TNAU pulse wonder at a rate of 1% during the peak flowering stage of blackgram and greengram.

#### 2.2.2. Traits Recorded

The morphological traits, *viz*., plant height and number of branches, given in plant^−1^, were recorded in 5 plants in each replication. The physiological parameters, *viz*., chlorophyll index (SPAD value), photosynthetic rate, stomatal conductance, and transpiration rate, were recorded in the control and for blackgram and greengram plants sprayed with TNAU pulse wonder. In each replication, the physiological traits were recorded in five plants. The chlorophyll index was measured for the leaves using a soil–plant analysis development meter (Minolta SPAD 502). Gas exchange parameters, *viz.,* photosynthetic rate (*P*n) (µmol CO_2_ m^−2^ s^−1^), stomatal conductance (*gs*) (mol H_2_O m^−2^ s^−1^), and transpiration rate (*E*) (mmol H_2_O m^−2^ s^−1^), were measured using a Portable Photosynthesis System (LI-6400 XT; LI-COR Inc., Lincoln, NE, USA). The measurements were recorded between 9.30 and 11.00 a.m. on sunny days in fully expanded leaves. The measurement was taken at a light intensity of 1500 µmol m^−2^ s^−1^ PAR, a leaf temperature of 30 °C, and a constant CO_2_ concentration of 410 ± 5 µmol CO_2_ mol^−1^ in the sample chamber when provided with buffer volume. Similarly, the yield and yield components, such as number of clusters plant^−1^, number of pods cluster ^−1^, number of seeds pod ^−1^, total number of pods plant^−1^, pod length, dry matter production, and seed yield, were recorded.

### 2.3. Groundnut

A field experiment was conducted at the Eastern block farmland, TNAU, Coimbatore, to evaluate the effect of TNAU groundnut-rich spray on groundnut variety CO 7. The experiment was carried out in an area of 4000 m^2^ with a plot size of 150 m^2^ containing 5000 plants by adopting a randomized block design with three replications. Sowing was performed in beds and channels.

#### 2.3.1. Treatment Imposition

Two treatments, *viz.*, control (unsprayed control) and foliar spraying with TNAU groundnut tich 1%, were imposed at the flowering and pod-filling stages. We used a total of 2 kg of groundnut-rich sprays per acre for each spray.

#### 2.3.2. Traits Recorded

Physiological traits, *viz*., chlorophyll index, leaf temperature, photosynthetic rate, stomatal conductance, and transpiration rate, were measured one week after the completion of groundnut-rich spraying twice. The parameters, namely chlorophyll index and gas exchange parameters, were recorded as described in Section 2.2.2. The leaf temperature was recorded in the three uniform-sized leaves using a hand-held infrared radiometer (Apogee, Santa Monica, CA, USA, MI 220). The yield and associated traits, *viz.*, number of pods plant^−1^, number of seeds plant^−1^, pod weight plant^−1^, seed weight plant^−1^, and shelling percentage, calculated as [seed weight/pod weight × 100], were assessed after harvesting the crop of 15 plants for each treatment.

### 2.4. Cotton

A field study was carried out in an area of 4000 m^2^ with a plot size of 500 m^2^ containing 2200 plants by adopting a randomized block design with three replications in order to evaluate the effect of TNAU cotton plus application at the flowering and boll development stages on the CO 17 cotton cultivar. On the 12th day after sowing, gap filling was carried out. Plant protection measures were followed as per the Crop Production Guide [34].

#### 2.4.1. Treatment Imposition

Two treatments were carried out, *viz.*, the control (unsprayed control) and foliar spray of 1.25% TNAU cotton plus at flowering and boll development stages.

#### 2.4.2. Traits Recorded

Physiological parameters, such as chlorophyll index and gas exchange parameters, were recorded as described in Section 2.2.2. The photochemical efficiency (F_v_/F_m_) was recorded using an OS30p+ fluorometer by acclimatizing the leaves in the dark for 30 min. The morpho-physiological, yield, and quality traits, such as leaf area (cm^2^ plant^−1^), number of sympodia per plant, total dry weight (g plant^−1^), number of bolls plant^−1^, seed cotton yield (g plant^−1^), yield per ha (quintal ha^−1^), fibre length (mm), and fibre strength (g tex^−1^), were assessed in five plants of each replication.

### 2.5. Sugarcane

A field experiment was conducted in an area of 4000 m^2^ with a plot size of 500 m^2^ containing 1200 plants by adopting a randomized block design with three replications to evaluate the effect of TNAU sugarcane booster in the CoC 25 sugarcane variety.

#### 2.5.1. Treatment Imposition

Two treatments we performed, *viz.*, the control (unsprayed control) and a foliar spray of TNAU sugarcane booster. On the 45th DAP, 0.5% of the TNAU Sugarcane booster was sprayed. Similarly, at the 60th and 75th DAP, 0.75% and 1% of TNAU sugarcane booster, respectively, were sprayed.

#### 2.5.2. Traits Recorded

Morphological traits, *viz*., plant height (cm), number of tillers ha^−1^, number of millable cane ha^−1^, number of nodes cane^−1^, length of internode (cm), leaf area (cm^2^ plant^−1^), and leaf area index, were recorded. Apart from that, the total chlorophyll content (mg g^−1^) was estimated using 80% acetone, as described by Arnon [35]. The yield and associated traits, namely, cane length (cm), cane girth (cm), weight of single cane (g plant^−1^), cane yield (t ha^−1^), sugar yield (t ha^−1^), and commercial cane sugar content (%), were recorded at harvest.

### 2.6. Coconut

A field experiment was conducted in an area of one acre to evaluate the effect of TNAU coconut tonic on 10-year-old East Coast tall by adopting randomized block design with three replications. Fifty well-grown coconut trees were selected in the Tamil Nadu Agricultural Farm, out of which 25 palms were not given TNAU coconut tonic while the other 25 palms were fed with TNAU coconut tonic through the root. The TNAU coconut tonic was used for root feeding at a rate of 200 mL palm^−1^. A small pit was made 1.5 metres away from the main trunk, an active absorbing root of pencil thickness was selected, and a slanting cut was made. The TNAU coconut tonic (200 mL palm^−1^) was taken in a polythene bag, and the cut end of the root was dipped into the solution; the polythene bag was tied along with the root using a thread and left until the solution was absorbed. Coconut tonic was applied at six-month intervals, and the yield was recorded after 12 months.

### 2.7. Statistical Analysis

All the field experiments were conducted in a randomized block design in a one-acre field. The data were analyzed using the PROC GLM procedure of SAS 9.4. The means were separated by using the LSD test at a probability level of 0.05.

## 3. Results and Discussion

### 3.1. Maize

The effects of the foliar spraying of TNAU maize maxim on maize yield and yield attributes are presented in Table 5. The application of TNAU maize maxim significantly (*p* < 0.05) increased the 100-grain weight (37%) and grain yield per plant (24%) compared to the control treatment. However, the foliar spraying of TNAU maize maxim onto maize did not influence the cob length (cm), cob width (cm), number of seed rows cob^−1,^ and number of grains, given in row^−1^ (Table 5), because the spraying was carried out after the number of grains, given in cob^−1^, was decided. The enhancement of grain yield by the foliar application of maize maxim was associated with increased 100-grain weight. The increase in seed weight could be associated with salicylic acid and boron [36]. The increased uptake of other nutrients, the production of photoassimilate, and the translocation of carbohydrates via K and Zn, which are present in the TNAU maize maxim formulation, improved grain yield [37,38].

### 3.2. Greengram and Blackgram

In greengram, the foliar application of TNAU pulse wonder at the rate of 1% during peak flowering stage significantly (*p* < 0.05) increased plant height (17%) and dry matter production (39%) compared to the control plants (Table 6). Similarly, there was a significant increase in chlorophyll content (SPAD value, 8%), photosynthetic rate (12%), and stomatal conductance (22%) compared to the control plants (Table 6). However, the transpiration rate (8%) decreased compared to the control. The foliar spraying of pulse wonder at 1% significantly increased the number of pods cluster ^−1^ (37%), number of seeds pod^−1^ (12%), total number of pods plant^−1^ (64%), pod length (11%), and seed yield (19%) compared to the control (Table 7).

In blackgram, the foliar application of TNAU pulse wonder at 1% at the peak flowering stage significantly (*p* < 0.05) increased plant height (10%) and dry matter production (20%) compared to the control (Table 8). Similarly, we observed significant increases in chlorophyll content (SPAD value, 17%), photosynthetic rate (21%), stomatal conductance (68%), and transpiration rate (36%) compared to the control (Table 8). The foliar spraying of pulse wonder at 1% significantly increased the total number of pods plant^−1^ (47%), pod length (5%), and seed yield ha^−1^ (19%) compared to the control (Table 9). TNAU pulse wonder contains nitrogen, NAA, and salicylic acid. The foliar application of nitrogen and auxin might have increased reproductive success, which was observed as an increased number of pods plant^−1^. Similar to the results of the present study, Sharief et al. [38] reported that the foliar application of NAA up to 60 mg L^−1^ decreased flower shedding by 60–80%. Apart from this, the potassium and salicylic acid present in TNAU pulse wonder might be involved in the carbohydrate translocation from the leaf and stem to developing seeds, as evidenced by increased seed weight [39]. The application of nutrients leads to normal cell division and differentiation processes and facilitates the movement of photosynthates to the sink tissues, resulting in higher yields and enhanced dry matter production [40].

### 3.3. Groundnut

The foliar application of TNAU groundnut-rich solution at 1% during the flowering and pod-filling stages significantly increased the chlorophyll index (39%), photosynthetic rate (21%), stomatal conductance (28%), and transpiration rate (22%) and decreased the canopy temperature by −0.88 °C compared to the control (Table 10). TNAU groundnut-rich solution contains potassium which might improve the stomatal opening, resulting in an increased photosynthetic rate [41], and this was observed in the present study (Table 10). Similarly, the application of inorganic fertilizers might have increased the availability of potassium, iron, magnesium and sulphur, resulting in increased chlorophyll content, photosynthetic rate, stomatal conductance, and transpiration rate [42], and a higher rate of translocation of photoassimilate from the source to its sink [43]. Chitdeshwari et al. [44] demonstrated that in groundnut, the application of potassium during the flowering stage increased the number of flowers and pegs, and enhanced pod setting.

Similarly, the foliar application of 1% groundnut-rich solution significantly increased the number of pods plant^−1^ (75%), number of seeds plant^−1^ (60%), pod weight plant^−1^ (66%), seeds weight plant^−1^ (67%), and shelling percentage (23%) compared to the control (Table 11). The pod yield increased by 18%, and the kernel yield increased by 16% due to the application of 1% TNAU groundnut-rich solution (Table 11). TNAU groundnut-rich solution contains NAA. Adding this might have increased the pod-set percentage, thereby increasing the seed yield [45], as shown in Table 11.

### 3.4. Cotton

The foliar application of TNAU cotton plus during the flowering stage (1.25%) and boll development stage (1.25%) significantly increased the leaf area (8%) and total dry weight (14%) compared to the control (Table 12). There were similar occurrences for the chlorophyll index (SPAD value; 42%), photochemical efficiency (16%), photosynthetic rate (15%), and stomatal conductance (13%) compared to the control (Table 13). TNAU cotton plus contains K and Mg, these nutrients might enhance photosynthetic activity and improve dry matter partitioning [46,47]. The foliar application of nitrogen-containing formulations like TNAU cotton plus might have increased the activities of photosynthetic carbon assimilation enzymes (Rubisco), leading to a higher photosynthetic rate and cotton yield [48,49], Table 13.

The photochemical efficiency (F_v_/F_m_) is an important indicator of whether plants are under stress; a value above 0.8 indicates that the plants are not under stress, and a slight decline in the value of this parameter may correspond to a light protection mechanism. Plants sprayed with TNAU cotton plus had a higher value of the F_v_/F_m_ ratio, indicating a decrease in the photoinhibition and increase in PSII activity [50].

Likewise, the foliar application of TNAU cotton plus also significantly increased the number of bolls (plant^−1^; 11%), seed cotton yield (g plant^−1^; 16%), and yield (ha^−1^; 31%), and quality traits, *viz*., fibre length (3%) and fibre strength (8%) (Table 14). The application of cotton plus enhanced the leaf area, which may be associated with the presence of N, K, NAA, and salicylic acid in the formulation, which directly correlates with the photosynthetic rate [51]. The increase in cotton yield may be due to a reduction in flower and boll shedding and increased boll weight. The increased flower and pod set percentage may be associated with NAA, because it is present in TNAU cotton plus. Varma et al. [52] reported that the boll weight and seed cotton yield increased linearly due to the foliar application of 0.4 per cent urea with 0.4 per cent ZnSO_4_. A similar observation upon the foliar application of the TNAU cotton plus booster was reported by Sritharan et al. [53].

### 3.5. Sugarcane Booster

The foliar application of TNAU sugarcane booster at 45 (0.5%), 60 (0.75%), and 75 (1%) DAP significantly increased the number of tillers ha^−1^ (13%), number of millable cane ha^−1^ (14%), number of nodes cane^−1^ (31%), length of internodes (50%), and leaf area plant^−1^ (3%) compared to control (Table 15). Similarly, the application of sugarcane booster significantly increased the single cane weight (30%) and sugar yield (35%) compared to the control (Table 16). TNAU sugarcane booster contains nutrients like nitrogen, potassium, and Fe. A study indicated that the foliar application of micronutrients enhanced the chlorophyll content, resulting in a high photosynthetic rate [54]. The balanced use of micronutrients improves sugarcane juice quality in terms of sucrose content and increases yield. Overall, a balanced supply of micro- and macronutrients is indispensable for high-quality, long-term cane production [54]. The increase in sugarcane yield upon Zn application might be due to the participation of Zn in biosynthesis and in the activation of several enzymes and hormones, such as indole acetic acid, which might promote plant growth [55]. Thangavelu [56] observed that Zn fertilization, in addition to NPK, considerably increased the brix and pol % juice of cane as compared to the control. The present study revealed that the application of sugarcane booster increased the morphological and yield parameters of sugarcane, and this might be due to an adequate supply of phosphorus and other micronutrients, enhancing the leaf area, enzyme activity, cane yield, sugar content, and juice quality of sugarcane [57].

### 3.6. Coconut

The root feeding of TNAU coconut tonic at 200 mL palm^−1^ significantly increased the number of leaves (25%) and the number of spathes (50%) and reduced the button shedding (55%) (Table 17). Also, the root feeding of coconut tonic at 200 mL palm^−1^ at six-month intervals enhanced the single nut weight (36%) and nut yield (13%) (Table 17). The larger increase in the number of leaves palm^−1^, number of spathes palm^−1^, single nut weight^−1^ and nut yield palm^−1^ in TNAU coconut root-fed plants than that in control plants may be associated with the presence of macro- and micronutrients like B, Fe, Zn, and N in the TNAU coconut tonic. The decrease in button shedding seen in TNAU coconut tonic root-fed plants may be associated with NAA and salicylic acid, which are present in TNAU coconut tonic [58].

## 4. Conclusions

The food crop’s productivity must be increased to sustain the food security of the growing world population. Nutrient-efficient plants can increase crop yield through increased photosynthesis and yield components. It is well established that PGRs regulate a broad range of growth-related and developmental aspects of plants throughout their life cycle. Against this background, this study was conducted with crop-specific nutrient and PGRs formulations as crop boosters, and the result indicated that the foliar application of TNAU maize maxim at 1.5% at tassel initiation and grain-filling stages increased the grain yield by 24% compared to the control by improving the seed weight. Similarly, the foliar application of TNAU pulse wonder at 1% at the peak flowering stage improved the seed yield by 19% compared to the control through an increased pod-set percentage. The application of TNAU groundnut-rich solution at 1% at flowering and pod-filling stages of groundnut improved the pod yield by 18% and kernel yield by 16% compared to the control through increased pod-set percentage. The foliar application of TNAU cotton plus at 1.25% at flowering and boll development stages increased the lint yield ha^−1^ by 31% compared to the control through increased bolls plant^−1^. The application of TNAU sugarcane booster at 0.5% at 45 DAP, 0.75% at 60 DAP, and 1% at 75 DAP increased the single cane weight (30%) compared to the control, and this was primarily associated with the number of internodes and length of internodes. The root feeding of TNAU coconut tonic at 200 mL palm^−1^ at six-month intervals increased the nut yield (by 13% compared to the control) by increasing the single nut weight and number of nuts, given in palm^−1^, by reducing the button shedding. The adoption of TNAU crop boosters to increase crop productivity of these crops depends on the benefit-cost ratio (economic analysis) at the farm level. The effect of these crop-specific nutrients and PGRs formulations need to be assessed under different fertility levels of soils.

## Figures and Tables

**Table 1:** Information about the soil type, soil characteristics, irrigation schedule, fertilizer dose and schedule, spacing, and plot details of the field experiments.

Crop	Variety	Soil Chemical Composition and Texture	Irrigation Schedule	Recommended Dose of Fertilizers	Fertigation Schedule	Field Size (m^2^)	Plot Size (m^2^)	Spacing (cm)	Number of Plants Plot^−1^
Blackgram/ greengram	CO 8/ CO 8	Soil texture—clay loam with available nitrogen (22 kg ha^−1^), phosphorus (17 kg ha^−1^), and potassium (420 kg ha^−1^). pH—8.73 and EC—0.16 dS m^−1^.	Once in 10 days	25 kg N + 50 kg P_2_O_5_ + 25 kg K_2_O + 40 kg S ha^−1^	Fertilizer is applied basally before sowing.	4000	500	30 × 10	16,000
Groundnut	CO 7	Soil texture—clay loam with available nitrogen (204 kg ha^−1^), phosphorous (28 kg ha^−1^), potassium (858 kg ha^−1^). pH—7.8 and EC—0.23 dS m^−1^.	Once in 10 days	25 kg N + 50 kg P_2_O_5_ + 75 kg K_2_O + Gypsum 80 kg ha^−1^ as soil application at 45th day after sowing (DAS)	P_2_O_5_ fertilizer is applied as basal before sowing. N and K are applied in three splits, *viz.*, 50% N and K as basal + 25% N and K at 20 DAS + 25% N and K at 45 DAS.	4000	150	30 × 10	5000
Cotton	CO 17	Soil texture—sandy clay loamy; available nitrogen (159 kg ha^−1^), phosphorus (20 kg ha^−1^), potassium (448 kg ha^−1^); pH—8.92; and EC—2.5 dS m^−1^	Once in 10 days	60 kg N + 30 kg P_2_O_5_ + 30 kg K_2_O kg ha^−1^	In total, 50 per cent of N and K; full dose of P_2_O_5_ fertilizers was applied as basal before sowing, and the remaining 50 per cent N and K was applied to plants at 40–45 DAS.	4000	500	75 × 30	2200
Maize	Co 8	Soil texture—sandy clay loam; available nitrogen (159 kg ha^−1^), phosphorus (20 kg ha^−1^), potassium (448 kg ha^−1^); pH—8.92 and EC—2.5 dS m^−1^	Once in 10 days	135 kg N + 62.5 kg P + 50 Kg K_2_O ha^−1^	Overall, 1/4 dose of N; full dose of P_2_O_5_ and K_2_O were applied as basal before sowing. Overally, 1/4 dose of N was applied on the 25th DAS. The last dose (1/4) of N fertilizer was applied on 45th DAS.	4000	500	60 × 25	3300
Sugarcane	COC 25	Soil texture—sandy loam with available nitrogen (73 kg ha^−1^), phosphorus (11 kg ha^−1^), and potassium (120 kg ha^−1^). pH—7.6 and EC—0.12 dS m^−1^.	Once in 10 days	300 kg N + 100 kg P_2_O_5_ + 200 Kg K_2_O ha^−1^	N&P fertilizers are applied in equal splits at 30, 60, and 90 DAS. K fertilizer was applied during planting.	4000	500	90 × 45	1200
Coconut	East Coast Tall	Soil texture—sandy clay loam; available nitrogen (159 kg ha^−1^), phosphorus (20 kg ha^−1^), potassium (448 kg ha^−1^); pH—8.92 and EC—2.5 dS m^−1^	Once in 30 days	560 g N + 320 g P_2_O_5_ + 1200 g K_2_O palm^−1^	The fertilizers were applied in two equally split doses during June–July and November–December, respectively.	4000	1500	750 × 750	75

**Table 2:** The methodology followed for soil nutrient and protein estimation.

Nutrient	Method Used	Reagents Used	Grade & Purity	Brand	Instrument Used for Estimating the Nutrient Content	Make and Model	Reference
Nitrogen	Alkaline permang-anate method	0.32% KMnO_4_	AR; 99.5%	SRL Chemicals, Mumbai, India	-	-	[29]
		2.5% NaOH	ACS; 97%				
		2% boric acid	AR; 99.5%				
		0.5 N H_2_SO_4_	ICP; 95%	Thermo Fisher Scientific (Waltham, MA, USA)			
		0.5% bromocresol green	AR	SRL Chemicals, India			
		0.1% methyl red	ACS; 95%				
		Ethyl alcohol	99.9%	Thermo Fisher Scientific			
Phosphorous	Olsen’s method	0.5 M sodium bicarbonate	ACS; 99.7%	SRL Chemicals, India	Spectrophotometer	Shimadzu; UV-1800	[30]
		Darco G 60	1.8 g mL^−1^	Fisher Scientific			
		Ascorbic acid	AR; 99.7%	SRL Chemicals, India			
		1.5% ammonium molybdate	AR; 99%				
		10% stannous chloride	AR; 99%				
		100 ppm potassium dihydrogen phosphate	AR; 99.5%				
		H_2_SO_4_	ICP 95%	Thermo Fisher Scientific			
		HCl	32%				
Potassium	Neutral normal ammonium acetate method	1N ammonium acetate	AR; 98%	SRL Chemicals, India	Flame photometer	Elico; CL 378	[31]
		Acetic acid	Extra pure; 99.5%				
		1000 ppm potassium chloride	ACS; 99.5%				
Total Soluble Protein	Lowry’s method	2% sodium carbonate	AR; 99.5%	SRL Chemicals, India	Spectrophotometer	Shimadzu; UV-1800	[32]
		0.5% copper sulphate	AR; 99.5%				
		1% sodium potassium tartrate	AR; 99%				
		Folin–Ciocalteau reagent	AR				
		Bovine serum albumin 200 µg protein/mL	AR; 98%	Sigma-Aldrich, India (St. Louis, MO, USA)			
pH	Jackson method	Standard 4, 7 and 9.2 pH solutions	-	Thermo Fisher Scientific, Inc., Singapore	pH meter	EUTECH instruments	[33]
EC		0.01 N potassium chloride	ACS; 99.5%	SRL Chemicals, India	EC meter		

**Table 3:** The stage of application of TNAU crop-specific boosters.

Crop	Stage of Spray	BBCH Scale	Concentration of TNAU-Crop-Specific Booster Applied
Maize	Tasseling and silking stage	5.51—beginning of tassel emergence: tassel detectable at the top of the stem 7.71—beginning of grain development: kernels at blister stage, about 16% dry matter	1.5% TNAU maize maxim twice at tassel initiation and grain filling, respectively
Blackgram/greengram	Peak flowering	6.65—Full flowering: 50% of flowers open	1% TNAU pulse wonder
Groundnut	Flowering and pegging	6.65—full flowering 7.77—main phase of pod development: continuation of pod filling	1% TNAU groundnut-rich slution twice at flowering and pod development stage, respectively
Cotton	Flowering and boll formation	6.65—full flowering 7.75—about 50% of bolls have attained their final size	1.25% TNAU cotton plus twice at flowering and boll formation, respectively
Sugarcane	Tillering and stem elongation stage	Stage 2: tillering and side shoots Stage 3: stem elongation	0.5% TNAU sugarcane booster at 45 DAP; 0.75% TNAU sugarcane booster at 60 DAP and 1% TNAU sugarcane booster at 70 DAP
Coconut	At the time of flowering	Flowering stage	Root feeding of TNAU coconut tonic at the rate of 200 mL palm^−1^

**Table 4:** Composition of TNAU crop-specific boosters used for foliar or root feeding.

Crop	TNAU Crop Booster	Composition
Maize	Maize maxim	FeSO_4_, ZnSO_4_, K_2_SO_4_, MgSO_4_, borax, and salicylic acid
Blackgram/greengram	Pulse wonder	KCl, mono-ammonium phosphate, FeSO_4_, urea, borax, salicylic acid, and NAA
Groundnut	Groundnut-rich	FeSO_4_, K_2_SO_4_, MgSO_4_, borax, and NAA
Cotton	Cotton plus	MgSO_4_, mono-ammonium phosphate, KCl, urea, borax, salicylic acid, and NAA
Sugarcane	Sugarcane booster	FeSO_4_, borax, K_2_SO_4_, and urea
Coconut	Coconut tonic	KCl, urea, ZnSO_4_, boric acid, FeSO_4_, MgSO_4_, MnSO_4_, CuSO_4_, NAA, salicylic acid, and sodium molybdate

**Table 5:** Effect of foliar application of TNAU maize maxim at 1.5% at tassel initiation and 1.5% at grain-filling stages on the yield and yield components of maize.

Treatments	Cob Weight (g)	Cob Length (cm)	Cob Width (cm)	Number of Seed Rows cob^−1^	Number of Grains Row^−1^	Total Grain Weight (g)	100-Grain Weight (g)	Grain Yield (g)
Control	166.63 ^a^	17.57 ^a^	14.70 ^a^	14.30 ^a^	28.20 ^a^	131.10 ^a^	26.90 ^b^	6312 ^b^
Maize maxim	224.16 ^a^	20.27 ^a^	14.87 ^a^	14.67 ^a^	34.67 ^a^	179.84 ^b^	42.71 ^a^	7862 ^a^
CD (0.05)	12.87 *	3.52 ^NS^	3.48 ^NS^	1.43 ^NS^	7.98 ^NS^	32.31 *	1.93 *	480.2 **

Note: Statistical significance is denoted by lowercase letters, indicating that the means with the same letters had no significant difference at *p* < 0.05. Critical difference (CD) with * and ** indicates significance at *p* < 0.05 and *p* < 0.01, respectively. NS—Non significance at *p* < 0.05.

**Table 6:** Effect of foliar application of TNAU pulse wonder at 1% at peak flowering stage on plant height (cm), total dry matter production (kg ha^−1^), chlorophyll content (SPAD units), and gas exchange traits of greengram.

Treatments	Plant Height (cm)	Total Dry Matter Production (kg ha^−1^)	Chlorophyll Content (SPAD Units)	Photosynthetic Rate (µmol m^−2^ s^−1^)	Stomatal Conductance (mol m^−2^ s^−1^)	Transpiration Rate (mmol m^−2^ s^−1^)
Control	39.90 ^b^	2532.00 ^b^	43.93 ^b^	41.88 ^b^	0.91 ^a^	14.11 ^a^
TNAU pulse wonder	47.00 ^a^	3012.00 ^a^	47.66 ^a^	47.24 ^a^	1.17 ^b^	12.96 ^b^
CD (0.05)	15.19 **	189.45 **	8.01 **	10.94 **	0.24 **	2.97 **

Note: Statistical significance is denoted by lowercase letters, indicating that the means with the same letters had no significant difference at *p* < 0.05. Critical difference (CD) with ** indicates significance at *p* < 0.01.

**Table 7:** Effect of foliar application of TNAU pulse wonder @ 1% at peak flowering stage on yield and component traits in greengram.

Treatments	Number of Pods Cluster^−1^	Number of Seeds Pod^−1^	Total Number of Pods Plant^−1^	Pod Length (cm)	Pod Weight (g plant^−1^)	Seed Weight (g plant^−1^)	Total Dry Matter Production (g plant^−1^)	100 Seed Weight (g plant^−1^)	Seed Yield (kg ha^−1^)
Control	5.10 ^b^	10.40 ^b^	25.00 ^b^	7.27 ^b^	8.51 ^b^	4.96 ^b^	23.15 ^b^	3.87 ^b^	892.0 ^b^
TNAU pulse wonder	7.00 ^a^	11.70 ^a^	41.00 ^a^	8.10 ^a^	24.95 ^a^	14.96 ^a^	32.23 ^a^	4.70 ^a^	1068.0 ^a^
CD (0.05)	4.55 **	5.88 *	10.43 **	1.97 ^NS^	12.21 **	9.19 **	5.90 **	1.64 **	301.3 **

Note: Statistical significance is denoted by lowercase letters, indicating that the means with the same letters had no significant difference at *p* < 0.05. Critical difference (CD) with * and ** indicates significance at *p* < 0.05 and *p* < 0.01, respectively. NS—Non significance at *p* < 0.05.

**Table 8:** Effect of foliar application of TNAU pulse wonder at 1% at peak flowering stage on plant height (cm), total dry matter production (kg ha^−1^), chlorophyll content (SPAD units), and gas exchange traits of blackgram.

Treatments	Plant Height (cm)	Total Dry Matter Production (kg ha^−1^)	Chlorophyll Content (SPAD Units)	Photosynthetic Rate (µmol m^−2^s^−1^)	Stomatal Conductance (mol m^−2^ s^−1^)	Transpiration Rate (mmol m^−2^ s^−1^)
Control	23.5 ^b^	2068 ^b^	46.13 ^b^	38.13 ^b^	0.52 ^b^	8.98 ^b^
TNAU pulse wonder	26.0 ^a^	2499 ^a^	53.89 ^a^	46.47 ^a^	0.87 ^a^	12.29 ^a^
CD (0.05)	10.8 *	377.4 **	5.5 **	8.6 **	0.2 **	7.0 **

Note: Statistical significance is denoted by lowercase letters, indicating that the means with the same letters had no significant difference at *p* < 0.05. Critical difference (CD) with * and ** indicates significance at *p* < 0.05 and *p* < 0.01, respectively.

**Table 9:** Effect of foliar application of TNAU pulse wonder at 1% at peak flowering stage on yield and component traits of blackgram.

Treatments	Number of Pods Cluster^−1^	Number of Seeds Pod^−1^	Total Number of Pods Plant^−1^	Pod Length (cm)	Pod Weight (g plant^−1^)	Seed Weight (g plant^−1^)	Total Dry Matter Production (g plant^−1^)	100 Seed Weight (g plant^−1^)	Seed Yield (kg ha^−1^)
Control	4.70 ^a^	7.10 ^a^	20.40 ^b^	4.95 ^a^	8.92 ^b^	4.69 ^b^	7.93 ^b^	4.90 ^b^	947 ^b^
TNAU pulse wonder	5.30 ^a^	7.50 ^a^	30.0 ^a^	5.20 ^a^	13.50 ^a^	6.92 ^a^	9.86 ^a^	6.10 ^a^	1128 ^a^
CD (0.05)	5.7 ^NS^	4.3 ^NS^	14.1 **	2.6 ^NS^	4.8 **	3.6 **	3.9 **	1.4 **	166.9 **

Note: Statistical significance is denoted by lowercase letters, indicating that the means with the same letters had no significant difference at *p* < 0.05. Critical difference (CD) with ** indicates significance at *p* < 0.01. NS—Non significance at *p* < 0.05.

**Table 10:** Effect of foliar application of TNAU groundnut-rich solution at 1% at flowering and pod-filling stages on chlorophyll content (SPAD units) and gas exchange traits of groundnut.

Treatments	Chlorophyll Index (SPAD Units)	Photosynthetic Rate (μmol CO_2_ m^−2^ s^−1^)	Stomatal Conductance (mol H_2_O m^−2^ s^−1^)	Transpirational Rate (mmol H_2_O m^−2^ s^−1^)	Leaf Temperature (°C)
Control	27.43 ^b^	34.06 ^b^	0.92 ^b^	11.63 ^b^	32.81 ^a^
Groundnut-rich	38.37 ^a^	41.25 ^a^	1.18 ^a^	14.27 ^a^	31.93 ^b^
CD (0.05)	0.58 ***	6.44 *	0.37 *	1.01 *	0.46 *

Note: Statistical significance is denoted by lowercase letters, indicating that the means with the same letters had no significant difference at *p* < 0.05. Critical difference (CD) with *, and *** indicates significance at *p* < 0.05, and *p* < 0.001, respectively.

**Table 11:** Effect of foliar application of TNAU groundnut-rich solution at 1% at flowering and pod-filling stages on groundnut.

Treatments	Number of Pods Plant^−1^	Number of Seeds Plant^−1^	Pod Weight (g plant^−1^)	Seeds Weight (g plant^−1^)	Shelling Percentage (%)	Pod Yield (kg ha^−1^)	Kernel Yield (kg ha^−1^)
Control	20.57 ^b^	31.86 ^b^	22.78 ^b^	12.87 ^b^	47 ^b^	2067 ^b^	1392 ^b^
Groundnut-rich	36.14 ^a^	51.29 ^a^	37.88 ^a^	21.54 ^a^	58 ^a^	2455 ^a^	1625 ^a^
CD (0.05)	7.93 **	13.28 *	9.28 *	4.93 *	1.54 ***	747.96 *	452.67 *

Note: Statistical significance is denoted by lowercase letters, indicating that the means with the same letters had no significant difference at *p* < 0.05. Critical difference (CD) with *, ** and *** indicates significance at *p* < 0.05, *p* < 0.01, and *p* < 0.001, respectively.

**Table 12:** Effect of foliar application of TNAU cotton plus at 1.25% at flowering and at boll development stages on leaf area (cm^2^ plant^−1^), number of sympodia plant^−1^, and total dry matter (g plant^−1^) of cotton.

Treatments	Leaf Area (cm^2^ plant^−1^)	Number of Sympodia per Plant	Total Dry Matter (g plant^−1^)
Control	5263.01 ^b^	14.60 ^b^	78.86 ^b^
Cotton Plus	5704.04 ^a^	16.40 ^a^	90.65 ^a^
CD (0.05)	219.21 **	3.2 *	10.52 *

Note: Statistical significance is denoted by lowercase letters, indicating that the means with the same letters had no significant difference at *p* < 0.05. Critical difference (CD) with * and ** indicates significance at *p* < 0.05 and *p* < 0.01, respectively.

**Table 13:** Effect of foliar application of TNAU cotton plus at 1.25% at flowering and at boll development stages on chlorophyll content (SPAD units) and gas exchange traits of cotton.

Treatments	Chlorophyll Index (SPAD Value)	Photochemical Efficiency (F_v_/F_m_ Ratio)	Photosynthetic Rate (μmol CO_2_ m^−2^ s^−1^)	Transpiration Rate (mmol H_2_O m^−2^ s^−1^)	Stomatal Conductance (mmol H_2_O m^−2^ s^−1^)
Control	38.10 ^b^	0.67 ^b^	27.44 ^b^	8.74 ^a^	0.34 ^b^
Cotton Plus	54.16 ^a^	0.77 ^a^	31.56 ^a^	7.71 ^b^	0.38 ^a^
CD (0.05)	2.73 **	6.83 *	1.65 **	0.95 *	4.37 *

Note: Statistical significance is denoted by lowercase letters, indicating that the means with the same letters had no significant difference at *p* < 0.05. Critical difference (CD) with * and ** indicates significance at *p* < 0.05 and *p* < 0.01, respectively.

**Table 14:** Effect of foliar application of TNAU cotton plus at 1.25% at flowering and at boll development stages on cotton yield and fibre quality of cotton.

Treatments	Number of Bolls Plant^−1^	Seed Cotton Yield (g plant^−1^)	Yield Per Ha (Quintal ha^−1^)	Fibre Length—UHML (mm)	Fibre Strength (g tex^−1^)
Control	25.4 ^b^	109.91 ^b^	14.65 ^b^	25.92 ^b^	24.64 ^b^
Cotton Plus	28.2 ^a^	127.83 ^a^	19.21 ^a^	26.84 ^a^	26.62 ^a^
CD (0.05)	2.21 *	8.62 *	2.81 *	0.55 **	0.73 **

Note: Statistical significance is denoted by lowercase letters, indicating that the means with the same letters had no significant difference at *p* < 0.05. Critical difference (CD) with * and ** indicates significance at *p* < 0.05 and *p* < 0.01, respectively.

**Table 15:** Effect of foliar application of TNAU sugarcane booster at 45 (0.5%), 60 (0.75%), and 75 (1%) days after planting on morphological and total chlorophyll content of sugarcane.

Treatments	Plant Height (cm)	Number of Tillers ha^−1^ 10^3^	Number of Millable Cane ha^−1^ (×10^3^)	Number of Nodes Cane^−1^	Length of Internode (cm)	Leaf Area (cm^2^ plant^−1^)
Control	329.0 ^a^	147.0 ^b^	122.3 ^b^	19.0 ^b^	6.20 ^b^	718.0 ^b^
Sugarcane booster	354.3 ^a^	167.3 ^a^	140.0 ^a^	25.0 ^a^	9.36 ^a^	740.4 ^a^
CD (0.05)	40 ^NS^	7.9 **	9.4 *	2.4 *	2.9 *	18.0 *

Note: Statistical significance is denoted by lowercase letters, indicating that the means with the same letters had no significant difference at *p* < 0.05. Critical difference (CD) with * and ** indicates significance at *p* < 0.05 and *p* < 0.01, respectively. NS—Non significance at *p* < 0.05.

**Table 16:** Effect of foliar application of TNAU sugarcane booster at 45 (0.5%), 60 (0.75%), and 75 (1%) days after planting on yield and yield traits of sugarcane.

Treatments	Cane Length (cm)	Cane Girth (cm)	Single Cane Weight (g plant^−1^)	Cane Yield (t ha^−1^)	Sugar Yield (t ha^−1^)	Commercial Cane Sugar Content (%)
Control	249.0 ^a^	2.13 ^a^	1237.3 ^b^	109.0 ^a^	12.0 ^b^	10.8 ^a^
Sugarcane booster	274.0 ^a^	2.45 ^a^	1611.3 ^a^	124.6 ^a^	16.3 ^a^	11.2 ^a^
CD (0.05)	12 *	0.8 ^NS^	148.6 **	10.1 *	1.2 **	2.1 ^NS^

Note: Statistical significance is denoted by lowercase letters, indicating that the means with the same letters had no significant difference at *p* < 0.05. Critical difference (CD) with * and ** indicates significance at *p* < 0.05 and *p* < 0.01, respectively. NS—Non significance at *p* < 0.05.

**Table 17:** Effect of root feeding of TNAU coconut tonic at 200 mL palm^−1^ on morphological and yield traits on coconut.

Treatments	Number of Leaves	Number of Spathes Plant^−1^	Number of Female Flowers Spathe^−1^	Button Shedding %	Single Nut Weight (g)	Nut yield (Numbers Tree^−1^)	Average Nut Yield (Numbers ha^−1^)
Control	28 ^b^	8 ^b^	112 ^b^	45 ^a^	720 ^b^	115 ^b^	20,125 ^b^
TNAU coconut tonic	35 ^a^	12 ^a^	163 ^a^	20 ^b^	980 ^a^	130 ^a^	22,775 ^a^
CD (0.05)	0.69 **	0.29 **	3.98 **	0.62 **	22.48 **	7.42 **	976.51 **

Note: Statistical significance is denoted by lowercase letters, indicating that the means with the same letters had no significant difference at *p* < 0.05. Critical difference (CD) with ** indicates significance at *p* < 0.01.

## Data Availability

The data used and presented in this paper are available upon request from the corresponding author. All the data were available on manuscript.

## Supplementary Materials

The following supporting information can be downloaded at: https://www.mdpi.com/article/10.3390/plants13111549/s1, Table S1: Weather parameters observed during the experimental period.
