# Supplementary material for: Sustaining the Yield of Maize, Blackgram, Greengram, Groundnut, Cotton, Sugarcane, and Coconut through the Application of Nutrients and Plant Growth Regulator Mixture
Source: Plants (Basel). 2024 Jun 4;13(11):1549. doi: 10.3390/plants13111549 (PMC11175130; doi:10.3390/plants13111549)
Supplement: Supplementary file 1 [file plants-13-01549-s001.zip › plants-2987296-supplementary.pdf]

Supplementary Table S1. Weather parameters observed during the experimental period.

Month	Std. Week	2020					2021					2022				
		Max. Temp	Min. Temp	RH (Morn.)	RH (Even.)	Rainfall	Max. Temp	Min. Temp	RH (Morn.)	RH (Even.)	Rainfall	Max. Temp	Min. Temp	RH (Morn.)	RH (Even.)	Rainfall
June	**22**	33.1	24.8	81.0	57.0	29.0	34.3	23.6	83.0	49.0	0.0	33.7	23.4	79.0	51.0	0.5
	**23**	32.7	24.3	81.0	54.0	0.5	33.7	24.1	81.0	49.0	0.0	34.4	24.1	83.0	49.0	0.0
	**24**	32.1	24.1	81.0	53.0	6.5	30.6	23.8	78.0	62.0	12.0	33.5	23.4	83.0	52.0	8.0
	**25**	32.4	23.9	80.0	54.0	1.5	32.1	23.9	78.0	55.0	2.0	32.2	23.6	82.0	50.0	0.5
July	**26**	32.8	23.6	84.0	55.0	14.0	32.7	23.0	82.0	52.0	1.0	31.1	23.5	78.0	56.0	5.5
	**27**	31.7	23.3	83.0	54.0	7.0	32.9	24.0	85.0	58.0	7.0	29.5	23.0	79.0	63.0	18.5
	**28**	31.3	23.0	86.0	64.0	10.5	30.4	23.4	80.0	64.0	5.5	28.8	22.9	81.0	66.0	21.0
	**29**	31.4	23.1	85.0	59.0	23.0	29.8	23.2	81.0	62.0	15.5	30.0	23.3	83.0	65.0	23.0
	**30**	32.4	23.5	83.0	60.0	43.0	31.0	23.9	77.0	59.0	2.5	31.1	23.2	87.0	55.0	13.0
August	**31**	29.9	23.1	82.0	60.0	26.0	32.4	23.0	82.0	51.0	0.0	30.7	23.0	86.0	64.0	33.8
	**32**	29.6	23.1	83.0	64.0	23.0	32.5	23.4	85.0	56.0	1.0	28.5	23.4	80.0	63.0	9.6
	**33**	30.2	23.8	82.0	57.0	0.0	31.2	22.7	85.0	54.0	11.3	32.3	23.0	85.0	54.0	0.0
	**34**	32.6	23.6	86.0	54.0	0.5	31.3	22.8	85.0	63.0	14.2	31.2	22.9	86.0	58.0	4.1
	**35**	33.4	22.8	85.0	51.0	35.0	31.1	22.9	85.0	60.0	16.0	30.4	22.8	87.0	65.0	10.5
September	**36**	31.4	23.4	86.0	62.0	43.0	29.9	22.8	82.0	60.0	9.0	31.0	23.2	83.0	59.0	7.5
	**37**	29.0	22.9	84.0	68.0	7.0	32.2	23.6	81.0	54.0	0.0	30.2	22.7	83.0	57.0	2.0
	**38**	29.3	23.2	80.0	65.0	32.5	33.2	23.3	85.0	55.0	3.0	32.2	22.0	83.0	46.0	0.0
	**39**	30.9	23.2	85.0	59.0	23.0	31.2	23.3	83.0	59.0	17.5	31.9	22.1	85.0	52.0	2.5
October	**40**	32.2	22.4	85.0	51.0	0.0	30.9	23.5	87.0	62.0	37.0	30.9	22.7	84.0	52.0	15.5
	**41**	30.8	23.4	80.0	57.0	21.5	30.9	23.3	83.0	65.0	11.5	30.6	22.6	85.0	59.0	30.5
	**42**	31.0	22.8	82.0	64.0	14.5	31.0	23.5	86.0	66.0	66.5	30.9	22.7	87.0	63.0	40.5
	**43**	32.4	22.5	82.0	45.0	0.0	30.3	22.7	86.0	64.0	86.0	30.8	21.5	85.0	53.0	5.0
	**44**	33.2	22.8	85.0	45.0	14.0	28.1	23.0	87.0	71.0	104.0	29.7	22.6	83.0	62.0	13.0
